# Evaluating anti-MRSA antibiotic stewardship with a focus on trends in consumption and resistance in a tertiary hospital in Alexandria, Egypt from 2019 to 2023

**DOI:** 10.1186/s13690-025-01614-3

**Published:** 2025-05-14

**Authors:** Ehab Elmongui, Adel Zaki, Amel Elsheredy, Asmaa Abd Elhameed

**Affiliations:** 1 Department of Biomedical Informatics and Medical Statistics, Medical Research Institute, Alexandria University, Alexandria, Egypt; 2 Department of Microbiology, Medical Research Institute, Alexandria University, Alexandria, Egypt

**Keywords:** Antimicrobial resistance (AMR), Methicillin-resistant *Staphylococcus* species (MRSS), Linezolid, Vancomycin, Teicoplanin, Antibiotic consumption, Antimicrobial stewardship

## Abstract

**Background:**

The global rise of antimicrobial resistance (AMR) threatens infection treatment. Methicillin-resistant *Staphylococcus* species (MRSS) are particularly challenging. This study examines the relative consumption of Linezolid (Reserve antibiotic) versus Vancomycin and Teicoplanin (Watch antibiotics) for MRSS, comparing trends with resistance patterns to optimize antibiotic use and combat AMR.

**Methods:**

This observational retrospective study analyzed trends in the consumption of Vancomycin, Linezolid, and Teicoplanin among all patients admitted to a tertiary hospital in Alexandria, Egypt from January 2019 to December 2023. The study compared these consumption patterns with resistance rates in *Staphylococcus* spp., including MRSS, from January 2020 to December 2023. Data on antibiotic consumption, expressed as defined daily doses per 1000 patient days (DDD/1000PD), were retrieved from pharmacy records, while resistance data were obtained from the WHONET database. Exploratory data analysis, including time series visualization and linear regression, assessed trends and the impact of COVID-19 on antibiotic use and resistance patterns.

**Results:**

Linezolid consumption increased significantly (β = 3.72, 95% CI: 0.50 to 6.94, *p* = 0.027), while Teicoplanin consumption also rose but to a lesser extent (β = 1.81, 95% CI: 1.02 to 2.60, *p* < 0.001). Vancomycin consumption remained stable (β = -0.31, 95% CI: -0.77 to 0.15, *p* = 0.184). Overall, Linezolid experienced an increase in usage that was 2.5 times steeper compared to the combined use of Vancomycin and Teicoplanin. The ICU surveillance data indicated that the days of Vancomycin therapy per 1000 patient days decreased significantly (β = -5.02, 95% CI: -6.79 to -3.25, *p* < 0.001). Methicillin resistance remained high ranging from 47.8 to 85.2%. Throughout the study period, resistance to the three antibiotics was higher than resistance rates reported in other published studies across Egypt while remained stable and comparable between the hospital and ICU. In the hospital, resistance ranged from 4 to 23.7% for Vancomycin, 6.3–28.6% for Linezolid, and 0–41.7% for Teicoplanin. In the ICU, Vancomycin resistance ranged from 5.5 to 34.2%, Linezolid from 11.4 to 41.2%, and Teicoplanin from 7.1 to 38.8%.

**Conclusions:**

This study underscores the urgent need for antimicrobial stewardship to reduce Linezolid overuse and address persistently high resistance rates against all anti-MRSA antibiotics.

**Supplementary Information:**

The online version contains supplementary material available at 10.1186/s13690-025-01614-3.

**Table:** 

Text box 1. Contributions to the literature
• Provides a comprehensive analysis of the temporal trends in the consumption of the main antibiotics used to treat Methicillin-resistant *Staphylococcus* species (MRSS) in the largest tertiary hospital in Alexandria, Egypt, highlighting the current stewardship outcomes.
• Links detailed antibiotic usage data with resistance rates in MRSS, underlining the unjustifiable increase in the use of the Reserve-list antibiotic, Linezolid.
• Supports the implementation of WHO’s AWaRe classification by offering empirical data essential for shaping local antimicrobial stewardship programs that adhere to global strategies.

## Background

The growing global burden of antimicrobial resistance (AMR) poses a serious threat to public health, jeopardizing the effective treatment of infectious diseases [1]. Among the pathogens of major concern is *Staphylococcus aureus*, a widespread bacterium capable of producing a wide spectrum of illnesses, from simple skin infections to life-threatening sepsis [2]. The rise and spread of Methicillin-resistant *Staphylococcus aureus* (MRSA), along with other Methicillin-resistant *Staphylococcus* species (MRSS), have intensified this challenge due to their inherent resistance to beta-lactam antibiotics [3].

In response to the escalating threat of AMR, the WHO Expert Committee on Selection and Use of Essential Medicines introduced the AWaRe categorization of antibiotics in 2017 as a tool to support antibiotic stewardship efforts at local, national, and global levels. This system classifies antibiotics into three categories—Access, Watch, and Reserve—based on their impact on antimicrobial resistance, emphasizing the importance of their appropriate use [4]. The Access antibiotics list comprises narrow-spectrum agents known for their favorable safety profiles, making them ideal candidates for de-escalating treatment regimens when susceptibility is confirmed by antibiotic sensitivity testing [5]. The Watch antibiotics list contains broad-spectrum agents typically advised as the initial empiric treatment for patients with severe clinical conditions or for infections caused by pathogens that are resistant to Access antibiotics [5]. The Reserve list consists of antibiotics that should be used as a last resort for treating multidrug-resistant infections [5]. The AWaRe classification is revised every two years and serves as a tool for tracking antibiotic usage, setting targets, and evaluating the impact of stewardship policies designed to optimize antibiotic use and reduce antimicrobial resistance [4].

Linezolid, classified as a Reserve-list antibiotic due to its efficacy against multidrug-resistant Gram-positive pathogens, has become a cornerstone in the management of severe MRSS infections [5]. However, the widespread use of Linezolid is commonly observed in clinical settings across both developed and developing countries, owing to its excellent pharmacodynamic and pharmacokinetic properties [6–9]. This raises concerns about the potential for rapid development of resistance, posing a future threat of losing one of the last remaining options for treating multidrug-resistant infections.

In contrast, Vancomycin and Teicoplanin are two glycopeptide agents while associated with adverse effects and requires frequent monitoring, have traditionally been considered as effective alternatives for MRSS infections. Teicoplanin is less nephrotoxic and requires less frequent monitoring than Vancomycin [10, 11]. These agents, belong to the WHO “Watch list” category of antibiotics, which require careful supervision but continue to be effective instruments in the antibacterial arsenal against MRSA and MRSS and should be considered in the local empiric antibiotic policies [5]. The optimal balance between the use of reserve agents like Linezolid and watch list antibiotics is crucial for preserving their efficacy and preventing the emergence of resistance.

Understanding the consumption patterns of these antibiotics is essential for effective antimicrobial stewardship. Studies have highlighted variations in antibiotic prescribing practices across different healthcare settings, with some reporting excessive use of Linezolid compared to watch list agents especially after the COVID-19 pandemic [12–14].

This study aims to contribute to the growing body of knowledge on antibiotic consumption and resistance patterns by examining trends in the use of Vancomycin, Teicoplanin, and Linezolid in a tertiary care hospital setting. By comparing antibiotic consumption data with resistance rates among *Staphylococcus* species, including MRSS, this research seeks to identify potential areas for improvement in antimicrobial stewardship practices and to inform targeted interventions to optimize antibiotic usage.

## Methods

### Study design

This observational retrospective study employed time series analysis to examine trends in the consumption of Vancomycin, Linezolid, and Teicoplanin among all patients admitted to a tertiary hospital in Alexandria, Egypt over a five-year period from January 2019 to December 2023. It also compared these trends with the percentage of resistance observed in *Staphylococcus* spp., including Methicillin resistance, during the period from January 2020 to December 2023. The study utilized aggregate-level data for Vancomycin, Linezolid, and Teicoplanin consumption from hospital pharmacy records, along with days of therapy data for Vancomycin from infection control surveillance records in the medical ICU.

### Study setting

The study took place at Gamal Abdel Naser Hospital, the primary tertiary health insurance facility in Egypt’s Northwest region. Situated in Alexandria, the hospital caters to the populations of Alexandria, El Beheira, and Marsa Matrouh governorates. It has 638 beds in total, including a 90-bed Intensive Care Unit (ICU) specialized in critical care, offering a wide range of healthcare services to the region.

### Data collection

Data on the consumption of Vancomycin, Teicoplanin, and Linezolid, as well as the percentage of resistance displayed by *Staphylococcus* spp. to these agents and the percentage of Methicillin-resistant *Staphylococcus* spp. (MRSS), were gathered and recorded in Microsoft Excel spreadsheets. Resistance data for the period from January 2020 to December 2023 were obtained from the WHONET database, on which the hospital depended for recording microbiologically and clinically documented infections, capturing antimicrobial sensitivity results according to the Clinical and Laboratory Standards Institute (CLSI) breakpoints, starting from January 2020.

For the hospital’s Vancomycin, Teicoplanin and Linezolid resistance, the number of isolates was sufficient to analyze data on quarterly basis while for ICU, the number of isolates allowed for biannual analysis (per semester).

The hospital’s consumption data for Vancomycin, Teicoplanin, and Linezolid were initially retrieved from the pharmacy records as the number of vials and tablets for Linezolid and then converted to defined daily doses per 1,000 patient days (DDD/1,000 PD) per quarter. For the ICU, only Vancomycin consumption data were available in the form of days of therapy per 1,000 patient-days (DOT/1,000 PD) as part of the infection control monthly surveillance activity. This data was then aggregated on a quarterly and semester basis.

### Data analysis

Exploratory data analysis included visualizing time series data for the consumption of Vancomycin, Teicoplanin, and Linezolid, as well as the percentage of resistance observed in *Staphylococcus* spp., and identifying trends using simple linear regression. Additionally, the analysis examined trends and levels of Linezolid and the combined consumption of Teicoplanin and Vancomycin, focusing on changes before and after Q1 2020 to assess the impact of COVID-19 case admissions to the hospital, which began in Q2 2020.

### Sample size

The study analyzed the complete records of Vancomycin, Teicoplanin and Linezolid resistance, encompassing a total of 1,344 *Staphylococcus* spp. isolates collected from the hospital, including 594 from the ICU, over a four-year period from January 2020 to December 2023.

## Results

### Trends in the total hospital consumption of linezolid, Teicoplanin, and Vancomycin

**Fig. 1:**
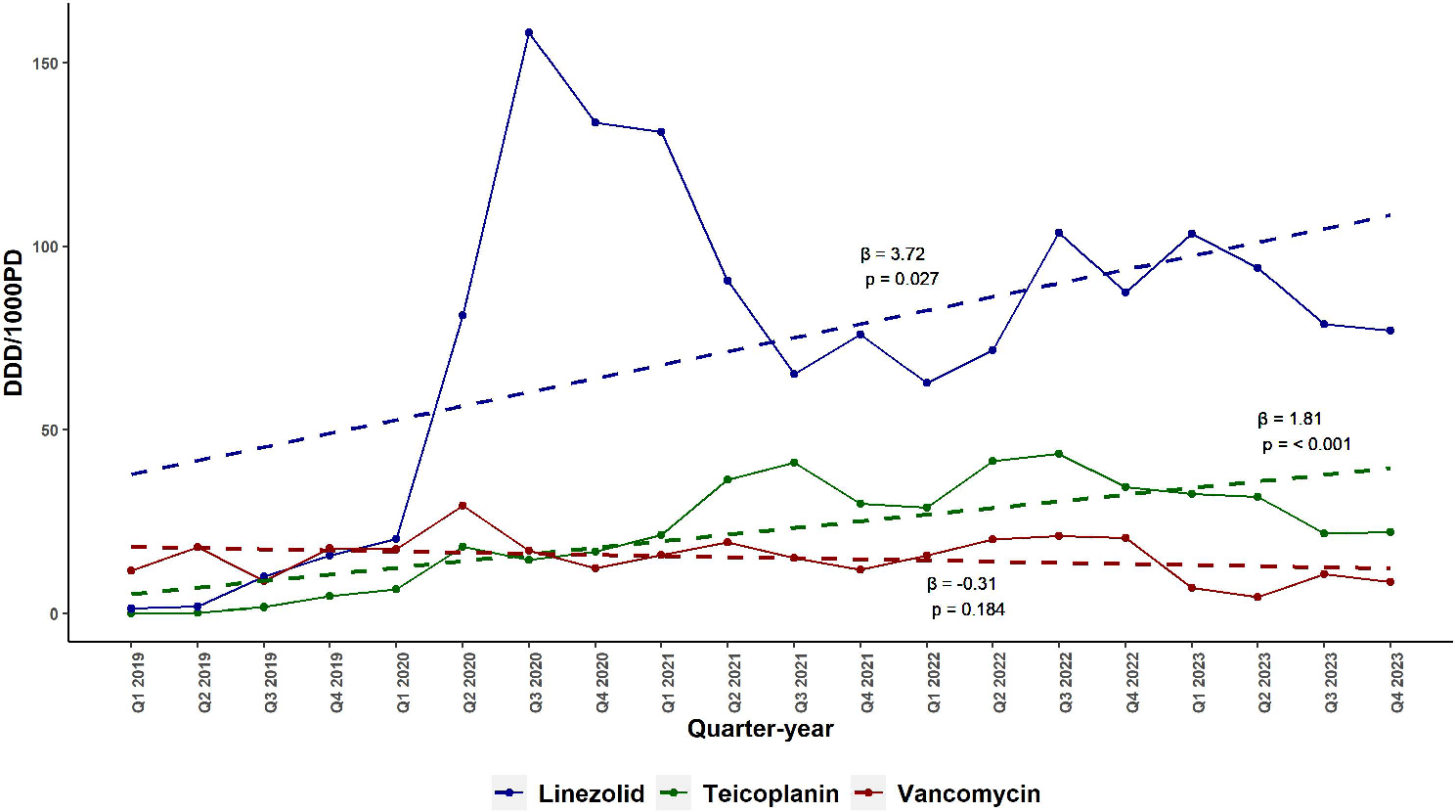
Quarterly Trends in the Hospital Consumption of Vancomycin, Linezolid, and Teicoplanin (DDD/1000 Patient-Days) in a Tertiary Hospital in Alexandria, Egypt from 2019 to 2023. This figure presents the quarterly consumption trends of three anti-MRSA antibiotics—Vancomycin, Linezolid, and Teicoplanin—in a tertiary hospital in Alexandria, Egypt from January 2019 to December 2023. Linezolid and Teicoplanin demonstrated rising usage trends, while Vancomycin consumption remained relatively stable. Abbreviations: DDD/1000PD, defined daily doses per 1000 patient-days; β, regression coefficient

Figure 1 depicts the quarterly consumption of Vancomycin, Linezolid, and Teicoplanin from January 2019 to December 2023. Overall, Linezolid consumption exhibited a steady increase (β = 3.72, 95% CI: 0.50 to 6.94, *p* = 0.027), while Teicoplanin consumption also increased significantly (β = 1.81, 95% CI: 1.02 to 2.60, *p* < 0.001). In contrast, Vancomycin consumption remained relatively stable throughout the study period (β = -0.31, 95% CI: -0.77 to 0.15, *p* = 0.184). Detailed consumption data are available in supplementary Table S1.

### Trends in the total hospital consumption of linezolid compared to the combined Teicoplanin and Vancomycin before and after COVID-19 pandemic

**Fig. 2:**
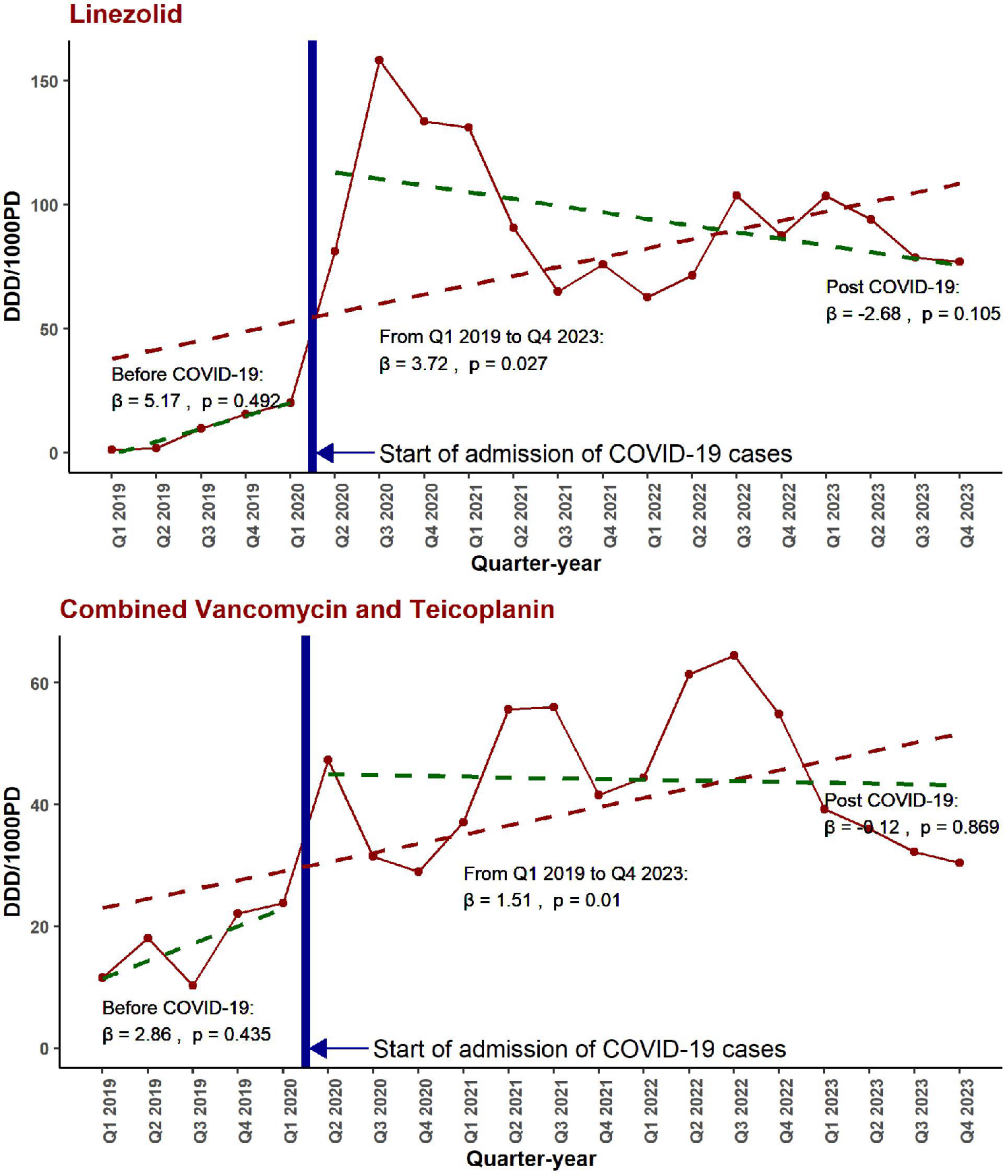
Segmented Quarterly Trends in Hospital Consumption of Linezolid and Combined Vancomycin/Teicoplanin Before and After the COVID-19 Pandemic in a Tertiary Hospital in Alexandria, Egypt from 2019 to 2023. This figure presents a segmented regression analysis of quarterly hospital consumption trends of Linezolid (top panel) and combined Vancomycin/Teicoplanin (bottom panel) before and after the onset of the COVID-19 pandemic in a tertiary hospital in Alexandria, Egypt. Vertical lines indicate the start of COVID-19 case admissions. Abbreviations: DDD/1000PD, defined daily doses per 1000 patient-days; β, regression coefficient

Figure 2; Table 1 both illustrate the quarterly trends in DDD/1000PD for Linezolid and combined Vancomycin/Teicoplanin before and after the COVID-19 pandemic. The analysis reveals that, prior to the pandemic, there was no notable trend in the usage of either Linezolid (β = 5.17, 95% CI: -10.40, 20.75, *p* = 0.492) or the combined Vancomycin/Teicoplanin (β = 2.86, 95% CI: -4.71, 10.43, *p* = 0.435). Following the onset of the pandemic, Linezolid usage experienced a sharp increase in consumption levels (β = 95.64, 95% CI: 49.03, 142.26, *p* < 0.001), though this change did not extend to a significant trend alteration (β = -2.68, 95% CI: -6.01 to 0.65, *p* = 0.105). Similarly, the combined Vancomycin/Teicoplanin usage saw a notable level change, nearing statistical significance (β = 22.15, 95% CI: -0.50, 44.79, *p* = 0.055), without a substantial trend modification (β = -1.12, 95% CI: -1.71 to 1.74, *p* = 0.869). Overall, the increase in Linezolid usage was 2.5 times steeper compared to Vancomycin/Teicoplanin (β: 1.51, 95% CI: 0.40 to 2.61, *p* = 0.01).

**Table 1:** Impact of the COVID-19 pandemic on level and quarterly trend changes in hospital consumption of linezolid and combined Vancomycin/Teicoplanin (DDD/1000 Patient-Days) in a tertiary hospital in Alexandria, Egypt from 2019 to 2023

Term	Hospital Linezolid consumption (DDD/1000PD)	Hospital combined Vancomycin/Teicoplanin consumption (DDD/1000PD
	β (95% CI)	β (95% CI)
(Intercept)	-5.76 (-57.43, 45.90)	8.60 (-16.5, 33.70)
Quarterly trend before COVID-19	5.17 (-10.40, 20.75)	2.86 (-4.71, 10.43)
Level change after COVID-19	95.64 (49.03, 142.26)*	22.15 (-0.50, 44.79)
Quarterly trend change after COVID-19	-7.86 (-23.71, 8.00)	-2.99 (-10.69, 4.72)

DDD: Defined Daily Doses. PD: Patient Days. β: regression coefficient representing change in DDD/1000 patient-days. CI: confidence interval. *Statistically significant (*p* < 0.05)

### Trends in the consumption of Vancomycin in ICU from the infection control surveillance records

**Fig. 3:**
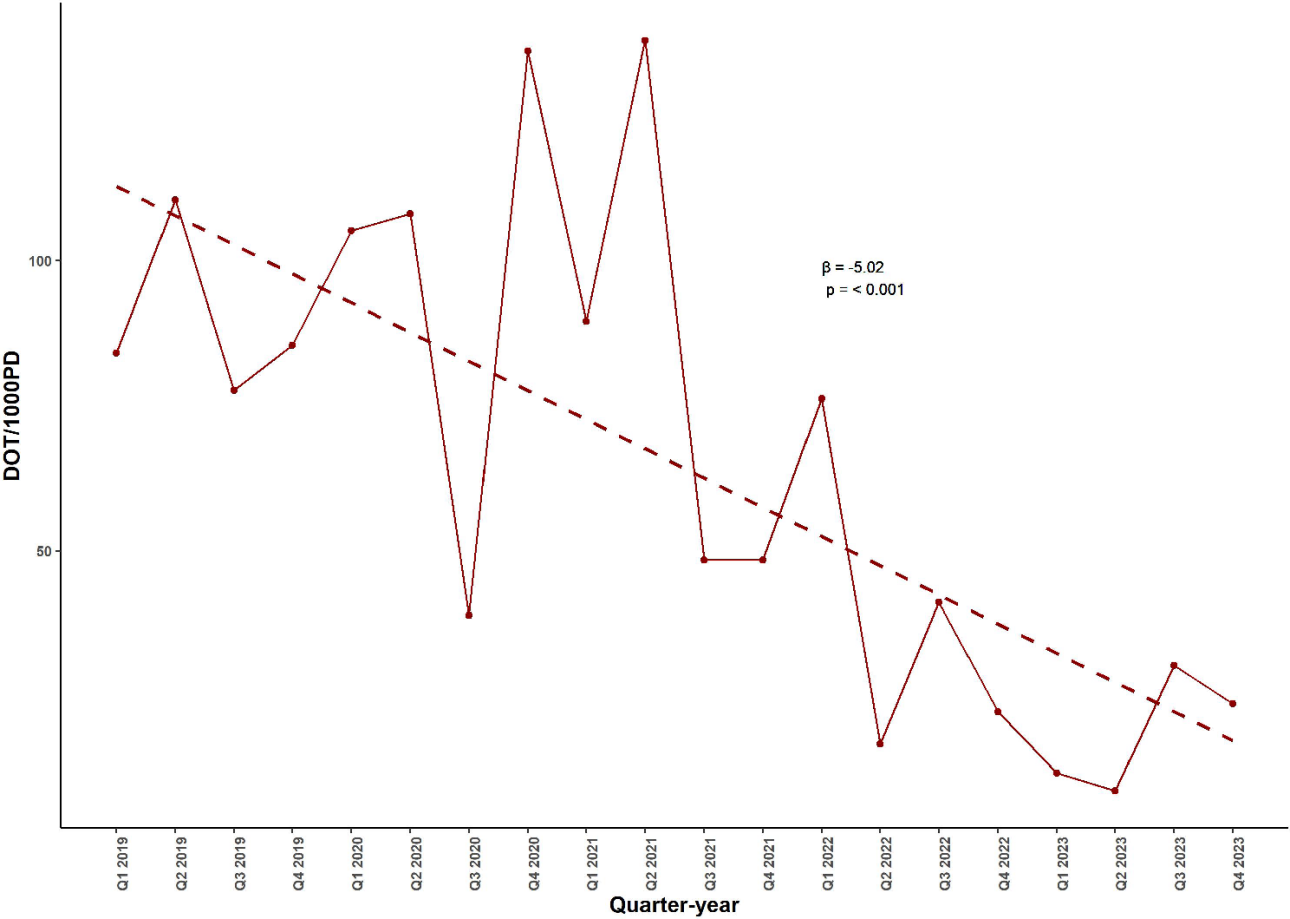
Quarterly Trend in ICU Vancomycin Consumption (DOT/1000 Patient-Days) in a Tertiary Hospital in Alexandria, Egypt from 2019 to 2023. This figure illustrates the quarterly trend in Vancomycin consumption within the intensive care unit (ICU) of a tertiary hospital in Alexandria, Egypt, from January 2019 to December 2023. The downward-sloping regression line (β = − 5.02) reflects a significant reduction in Vancomycin use over time. Abbreviations: DOT/1000PD, days of therapy per 1000 patient-days; β, regression coefficient

Figure 3 illustrates the quarterly ICU consumption of Vancomycin, calculated as days of therapy per 1000 patient days (DOT/1000PD), from January 2019 to December 2023, based on infection control surveillance records. A significant decreasing trend in Vancomycin consumption within the ICU was observed over the study period, as indicated by the negative linear regression line (β = -5.02, *p* < 0.001), suggesting a substantial reduction in Vancomycin utilization within the ICU.

### Exploratory data analysis for trends in resistance exhibited by Staphylococcus spp. Against linezolid, Teicoplanin, and Vancomycin in total hospital and ICU

**Fig. 4:**
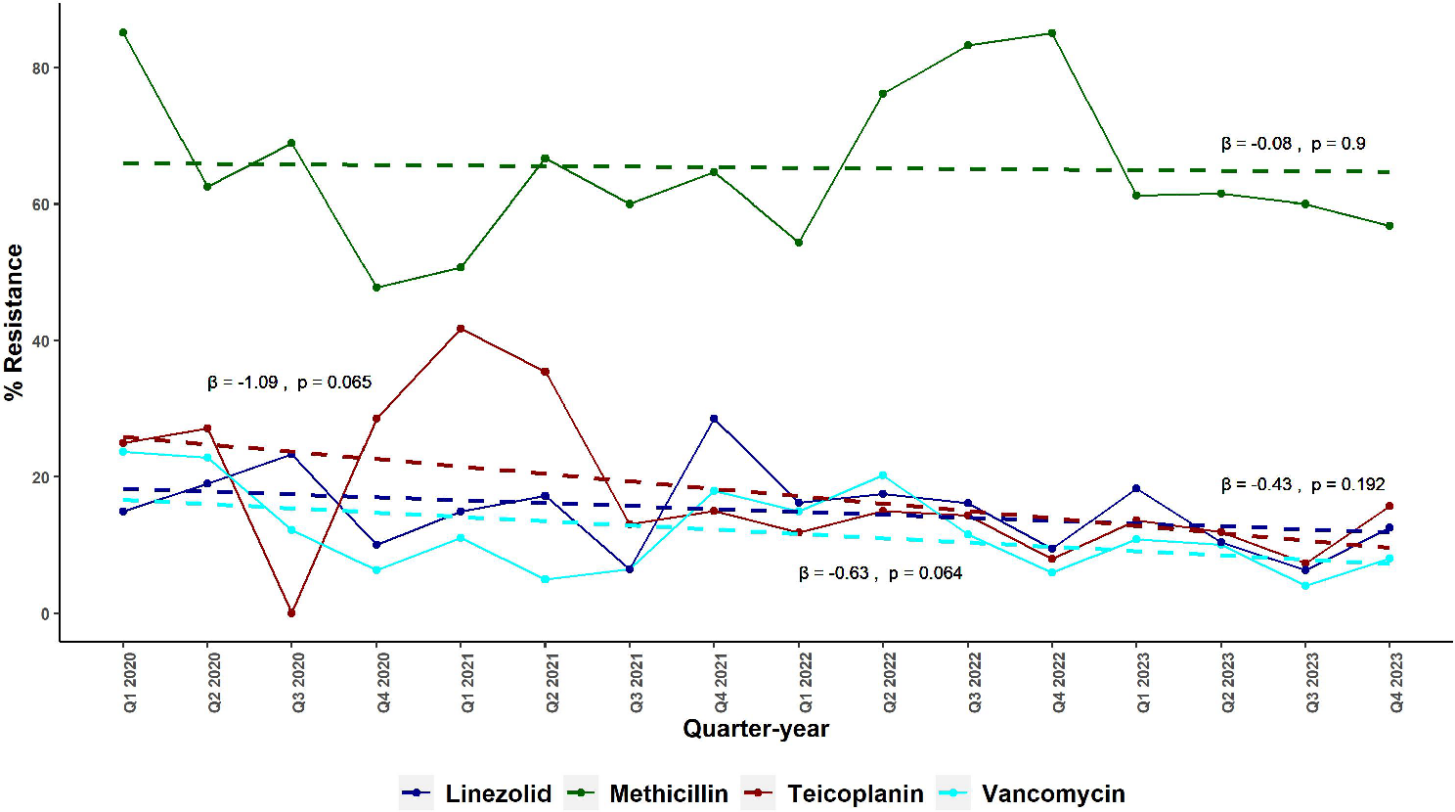
Quarterly Trends in Resistance of *Staphylococcus* spp. to Anti-MRSA Antibiotics in a Tertiary Hospital in Alexandria, Egypt from 2020 to 2023. This figure shows the quarterly resistance rates of Staphylococcus spp. to Methicillin, Vancomycin, Linezolid, and Teicoplanin in a tertiary hospital in Alexandria, Egypt, from January 2020 to December 2023. Methicillin resistance remained consistently high throughout the period, whereas resistance to Vancomycin, Linezolid, and Teicoplanin remained low and relatively stable. Abbreviations: β, regression coefficient; MRSA, methicillin-resistant *Staphylococcus aureus*

**Fig. 5:**
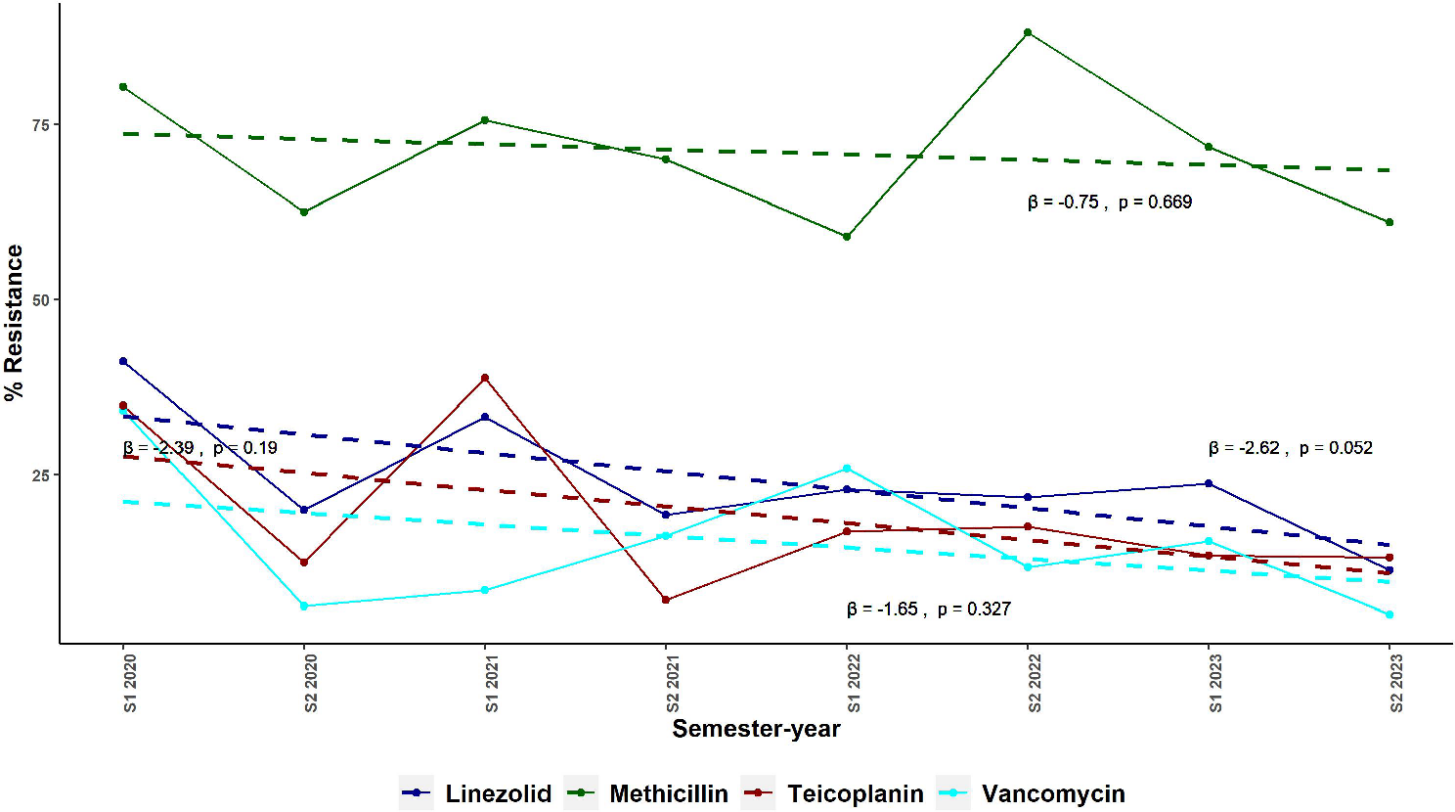
Semestral Trends in Resistance of *Staphylococcus* spp. to Anti-MRSA Antibiotics in the ICU of a Tertiary Hospital in Alexandria, Egypt from 2020 to 2023. This figure shows the semestral resistance rates of Staphylococcus spp. to Methicillin, Vancomycin, Linezolid, and Teicoplanin in the intensive care unit (ICU) of a tertiary hospital in Alexandria, Egypt, from January 2020 to December 2023. Methicillin resistance remained consistently high, similar to hospital-wide trends. Resistance to Vancomycin, Linezolid, and Teicoplanin showed more fluctuation, with Vancomycin resistance peaking in 2020 followed by a gradual decline. Abbreviations: β, regression coefficient; MRSA, methicillin-resistant *Staphylococcus aureus*

Figures 4 and 5 present the quarterly and semestral percentage of resistance of *Staphylococcus* spp. to Methicillin, Vancomycin, Linezolid, and Teicoplanin from January 2020 to December 2023 in the hospital and ICU, respectively. Methicillin resistance notably remained consistently high throughout the study period, with the hospital showing resistance rates ranging from 47.8% (95% CI: 33.4–62.2%) in Q4 2020 to 85.2% (95% CI: 77.5–92.9%) in Q1 2020. In the ICU, Methicillin resistance was similarly high, fluctuating between 59.0% (95% CI: 43.6–74.4%) in S1 2022 and 88.1% (95% CI: 81.2–95.0%) in S2 2022. Detailed data regarding % MRSS are available in supplementary Table S2.

In contrast, resistance to Vancomycin, Linezolid, and Teicoplanin in the total hospital and ICU remained relatively low and stable. For instance, Vancomycin resistance in the total hospital fluctuated between 4.0% (95% CI: 0.2–7.8%) in Q3 2023 and 23.7% (95% CI: 15.1–32.3%) in Q1 2020, while Linezolid resistance ranged from 6.3% (95% CI: 1.4–11.2%) in Q3 2023 to 28.6% (95% CI: 13.6–43.6%) in Q4 2021. Teicoplanin resistance showed a similar pattern, with the lowest resistance observed in Q3 2020 at 0.0% (95% CI: 0.0–0.0%) and the highest in Q1 2021 at 41.7% (95% CI: 13.8–69.6%), however, the highest resistance in Q1 2021 was based on a low number of tests (*n* = 12).

In the ICU, Vancomycin resistance showed a notable peak in S1 2020 at 34.2% (95% CI: 23.3–45.1%), followed by a significant drop to 6.3% (95% CI: 0.0–14.7%) in S2 2020. Linezolid resistance in the ICU ranged from 11.4% (95% CI: 4.4–18.4%) in S2 2023 to 41.2% (95% CI: 24.7–57.7%) in S1 2020. Teicoplanin resistance also varied, peaking at 38.8% (95% CI: 5.0–72.6%) in S1 2021 and reaching a low of 7.1% (95% CI: 0.0–20.6%) in S2 2021. Additionally, the figures show overlapping resistance rates for Vancomycin, Linezolid, and Teicoplanin in both the total hospital and the ICU, indicating comparable resistance levels among these agents over the study period. The detailed resistance data are available in supplementary tables S3 and S4.

## Discussion

The results of this study reveal a worrisome trend in antibiotic use at our institution, especially emphasizing the excessive use of Linezolid compared to Vancomycin and Teicoplanin for treating MRSS infections. This pattern is particularly alarming given that Linezolid is meant to be a last-line option in for multidrug resistant infections, as recommended by the WHO as a Reserve list antibiotic.

The steady rise in Linezolid use, despite the availability of other equally effective options like Vancomycin and Teicoplanin, suggests a deeper problem in antibiotic prescribing practices. This overreliance on Linezolid risks hastening antimicrobial resistance, potentially compromising the effectiveness of this vital drug for future infections.

The observed steeper increase in the Linezolid compared to the combined Vancomycin and Teicoplanin consumption following the COVID-19 pandemic is particularly noteworthy. While the exact reasons for this surge require further investigation, a plausible explanation might be the frequent need for monitoring and dosage adjustments with Vancomycin and Teicoplanin, particularly in ICU patients with renal impairment, a common concern. Additionally, the availability of cheaper Linezolid generics and its excellent oral bioavailability may have contributed to its increased preference, especially over Teicoplanin, which is less nephrotoxic and requires less frequent monitoring compared to Vancomycin, during this period.

The consistently very high rates of Methicillin resistance observed throughout the study period are particularly alarming and align with similar findings reported in other studies across Egypt. A National surveillance in Egypt carried out between 2012 and 2014 in 28 hospitals, including 91 ICUs revealed that Methicillin-resistant *Staphylococcus aureus* (MRSA) made up 78.9% of the *Staphylococcus aureus* isolates [15]. These elevated resistance rates suggest a significant and ongoing issue with Methicillin-resistant *Staphylococcus aureus* (MRSA) within institutions. The persistence of such high resistance levels over time underscores the need for enhanced infection control measures and rigorous antimicrobial stewardship practices to address and mitigate the spread of MRSA.

Despite remaining stable over the four years, the resistance rates observed in this study for Linezolid, Vancomycin, and Teicoplanin were exceptionally higher than those reported globally and locally post-COVID-19. This significant discrepancy is especially troubling, considering the typically lower resistance rates observed in other regions, indicating that local factors might be driving this increased resistance. A recent multicenter cross-sectional study by Shaimaa Abdelaziz Abdelmoneim et al. (2024) examined AMR trends before and after the COVID-19 pandemic across sixteen governorates in Egypt. The study found that in 2019, during the pre-COVID-19 period, the Linezolid resistance rate among 307 MRSA isolates was 0.3%, while in 2022, during the post-COVID-19 period, the resistance rate among 309 MRSA isolates dropped to 0% [16]. A systematic review and meta-analysis by Ahmed Azzam et al., which analyzed 64 studies conducted in Egypt up to October 2022, investigated the prevalence of MRSA among Staphylococcus aureus clinical isolates. The analysis revealed pooled resistance rates of 5% [95% CI: 2–8] for Linezolid and 9% [95% CI: 6–12] for Vancomycin among MRSA isolates [17].

Despite the relatively high resistance rates observed over the four years for Linezolid, Vancomycin, and Teicoplanin, the resistance levels remained stable and comparable among the three agents. This stability in resistance rates does not justify the increased use of Linezolid relative to the combined use of Vancomycin and Teicoplanin, nor does it account for the decreasing trend in Vancomycin consumption in DOT/1000PD within the ICU, as reflected in the infection control surveillance records. The uniformity in resistance suggests that the shift in prescribing practices is not driven by differences in effectiveness or resistance profiles, but rather by other factors such as cost or convenience, which warrants further investigation.

It is imperative to acknowledge the limitations of this study, particularly since AMR surveillance in the hospital only began in November 2019 with the implementation of the WHONET system for documenting susceptibility results [18]. The early stages of this system faced significant challenges, including budget constraints and a shortage of testing disks, which may have impacted the accuracy and comprehensiveness of the recorded data. First, the hospital resistance rates for Teicoplanin from Q3 2020 to Q4 2021 were based on testing fewer than 30 isolates, falling short of the CLSI-recommended minimum for inclusion in antibiograms [19]. A similar limitation applies to the ICU resistance rates from the second half of 2020 to the second half of 2021. Second, Vancomycin susceptibility testing in the study setting was conducted by agar diffusion, which may have influenced the accuracy of the results compared to more standardized and precise methods that are recommended by the CLSI for testing Vancomycin like broth microdilution. Third, before 2024, the study setting only provided aggregate resistance data for all pathogenic *Staphylococcus* spp. and did not differentiate between *Staphylococcus aureus* and coagulase-negative *Staphylococcus* spp., limiting the assessment of specific prevalence and resistance patterns. Fourth, The ICU consumption data used for examining ICU trends for Vancomycin consumption in DOT/1000PD came from infection control surveillance records, which may not capture the entire ICU population, potentially leading to incomplete data and bias in the analysis. Fifth, despite the limitations of relying on surveillance data, it is important to note that the infection control surveillance system in the ICU does not include Linezolid, a reserve list antibiotic, which further limits the comprehensiveness of the data. Sixth, the hospital data included all antibiotics consumed, recorded as total vials dispensed per month and not stratified by location due to its paper-based recording system. This limitation prevented the analysis of DDD/1000 patient-days for Teicoplanin and Linezolid within the ICU, with only DOT data available for Vancomycin from ICU-specific surveillance records.

Nevertheless, the findings of this study have important implications for antimicrobial stewardship programs. Targeted interventions aimed at reducing Linezolid consumption and promoting the appropriate use of Vancomycin and Teicoplanin are urgently needed. Reducing Linezolid consumption not only helps preserve its effectiveness but also prevents unnecessary complications associated with overuse. Despite its benefits, Linezolid is linked to side effects, as shown by a systematic review by Dan Zhang et al. in 2023, which included forty observational studies with 6,454 patients and found that 37% of those treated experienced thrombocytopenia [20]. Training programs for healthcare providers, along with guidelines and clinical decision support tools, can play a crucial role in promoting a culture of antimicrobial stewardship. Moreover, real-time monitoring of antibiotic usage by the guidelines-recommended DOT/1000PD metric and resistance trends is vital for assessing the effectiveness of interventions and detecting new threats as they arise.

## Conclusions

This study highlights the concerning indiscriminate overuse of Linezolid for MRSS infections, coupled with persistently high Methicillin resistance rates. These findings underscore the urgent need for effective antimicrobial stewardship programs. By reducing Linezolid consumption and promoting appropriate Vancomycin and Teicoplanin use for MRSS infections, we can mitigate AMR and safeguard the efficacy of this vital Reserve antibiotic. Targeted interventions, enhanced surveillance, and further research are essential to address this critical issue. The exceptionally high resistance rates against all anti-MRSA agents observed in this study warrant urgent and further investigation to understand the underlying causes and to develop effective strategies for combating this growing threat. While this study provides valuable baseline data, its impact on a broader audience may be enhanced by future research that includes results from specific antimicrobial stewardship interventions.

## Electronic supplementary material

Below is the link to the electronic supplementary material.

Supplementary Material 1

## Data Availability

The datasets used and/or analyzed during the current study are available from the corresponding author on reasonable request.

## Abbreviations

AMR: Antimicrobial resistance
AMS: Antimicrobial stewardship
CLSI: Clinical and Laboratory Standards Institute
COVID-19: Coronavirus disease 2019
DDD/1000PD: Defined daily doses per 1000 patient days
DOT/1000PD: Days of therapy per 1000 patient days
MRSA: Methicillin resistant Staphylococcus aureus
MRSS: Methicillin resistant Staphylococcus species
ICU: Intensive care unit
WHO: World health organization
