# Supplementary material for: Evaluating anti-MRSA antibiotic stewardship with a focus on trends in consumption and resistance in a tertiary hospital in Alexandria, Egypt from 2019 to 2023
Source: Arch Public Health. 2025 May 14;83:133. doi: 10.1186/s13690-025-01614-3 (PMC12076974; doi:10.1186/s13690-025-01614-3)
Supplement: Supplementary file 1 — Supplementary Material 1 [file 13690_2025_1614_MOESM1_ESM.docx]

**Supplementary materials**

| **S1: Detailed Vancomycin, Linezolid, and Teicoplanin consumption (DDD/1000PD) in the hospital and ICU Vancomycin (DOT/1000PD) from Jan 2019 to Dec 2023 by quarters** | | | | |
| --- | --- | --- | --- | --- |
| Quarter | Vancomycin (DDD/1000PD) | Linezolid (DDD/1000PD) | Teicoplanin (DDD/1000PD) | Vancomycin in ICU (DOT/1000PD) |
| Q1 2019 | 12 | 1 | 0 | 84 |
| Q2 2019 | 18 | 2 | 0 | 110 |
| Q3 2019 | 9 | 10 | 2 | 78 |
| Q4 2019 | 18 | 16 | 5 | 85 |
| Q1 2020 | 17 | 20 | 6 | 105 |
| Q2 2020 | 29 | 81 | 18 | 108 |
| Q3 2020 | 17 | 158 | 14 | 39 |
| Q4 2020 | 12 | 134 | 17 | 136 |
| Q1 2021 | 16 | 131 | 21 | 90 |
| Q2 2021 | 19 | 91 | 36 | 138 |
| Q3 2021 | 15 | 65 | 41 | 48 |
| Q4 2021 | 12 | 76 | 30 | 48 |
| Q1 2022 | 16 | 63 | 29 | 76 |
| Q2 2022 | 20 | 72 | 41 | 17 |
| Q3 2022 | 21 | 104 | 43 | 41 |
| Q4 2022 | 20 | 87 | 34 | 22 |
| Q1 2023 | 7 | 103 | 32 | 12 |
| Q2 2023 | 4 | 94 | 32 | 9 |
| Q3 2023 | 11 | 79 | 22 | 486 |
| Q4 2023 | 8 | 77 | 22 | 468 |

| **S2: Detailed % Methicillin resistance exhibited by *Staphylococcus* spp. from Jan 2020 to Dec 2023 by quarters in the hospital and by semester in the ICU** | | | | | | | | | | | |
| --- | --- | --- | --- | --- | --- | --- | --- | --- | --- | --- | --- |
| **Hospital** | | | | | | **ICU** | | | | | |
| **Quarter** | **Total isolates** | ***Staph.* spp. isolates** | ***Staph.* spp. Isolation rate** | **n** | **% Methicillin resistance**  **(95% CI)** | **Semester** | **Total isolates** | ***Staph.* spp. isolates** | ***Staph.* spp. Isolation rate** | **n** | **% Methicillin resistance**  **(95% CI)** |
| Q1 2020 | 398 | 101 | 25.4% | 81 | 85.2% (77.5% to 92.9%) | S1 2020 | 356 | 78 | 21.9% | 71 | 80.3% (71.0% to 89.6%) |
| Q2 2020 | 233 | 58 | 24.9% | 56 | 62.5% (49.8% to 75.2%) | S2 2020 | 167 | 36 | 21.6% | 32 | 62.5% (45.7% to 79.3%) |
| Q3 2020 | 219 | 49 | 22.4% | 45 | 68.9% (55.4% to 82.4%) | S1 2021 | 193 | 49 | 25.4% | 45 | 75.6% (63.1% to 88.1%) |
| Q4 2020 | 230 | 55 | 23.9% | 46 | 47.8% (33.4% to 62.2%) | S2 2021 | 397 | 85 | 21.4% | 80 | 70.0% (60.0% to 80.0%) |
| Q1 2021 | 283 | 79 | 27.9% | 73 | 50.7% (39.2% to 62.2%) | S1 2022 | 398 | 85 | 21.4% | 39 | 59.0% (43.6% to 74.4%) |
| Q2 2021 | 232 | 69 | 29.7% | 63 | 66.7% (55.1% to 78.3%) | S2 2022 | 708 | 90 | 12.7% | 84 | 88.1% (81.2% to 95.0%) |
| Q3 2021 | 370 | 85 | 23.0% | 80 | 60.0% (49.3% to 70.7%) | S1 2023 | 444 | 86 | 19.4% | 53 | 71.7% (59.6% to 83.8%) |
| Q4 2021 | 354 | 86 | 24.3% | 85 | 64.7% (54.5% to 74.9%) | S2 2023 | 460 | 85 | 18.5% | 41 | 61.0% (46.1% to 75.9%) |
| Q1 2022 | 363 | 91 | 25.1% | 68 | 54.4% (42.6% to 66.2%) |  |  |  |  |  |  |
| Q2 2022 | 405 | 89 | 22.0% | 21 | 76.2% (58.0% to 94.4%) |  |  |  |  |  |  |
| Q3 2022 | 565 | 99 | 17.5% | 90 | 83.3% (75.6% to 91.0%) |  |  |  |  |  |  |
| Q4 2022 | 661 | 107 | 16.2% | 101 | 85.1% (78.2% to 92.0%) |  |  |  |  |  |  |
| Q1 2023 | 485 | 99 | 20.4% | 85 | 61.2% (50.8% to 71.6%) |  |  |  |  |  |  |
| Q2 2023 | 382 | 76 | 19.9% | 13 | 61.5% (35.0% to 88.0%) |  |  |  |  |  |  |
| Q3 2023 | 486 | 104 | 21.4% | 14 | 60.0% (34.3% to 85.7%) |  |  |  |  |  |  |
| Q4 2023 | 468 | 97 | 20.7% | 81 | 56.8% (46.0% to 67.6%) |  |  |  |  |  |  |
| **Total** | **6,134** | **1,344** |  |  |  | **Total** | **3,123** | **594** |  |  |  |

| **S3: Detailed Vancomycin, Linezolid, and Teicoplanin % resistance exhibited by Staphylococcus spp. in the hospital from Jan 2020 to Dec 2023 by quarters** | | | | | | | | | |
| --- | --- | --- | --- | --- | --- | --- | --- | --- | --- |
|  |  |  |  | Vancomycin | | Linezolid | | Teicoplanin | |
| Quarter | Total isolates | Number of *Staph.* spp. isolates | *Staph.* spp. Isolation rate | n | % R  (95% CI) | n | % R  (95% CI) | n | % R  (95% CI) |
| Q1 2020 | 398 | 101 | 25.4% | 93 | 23.7% (15.1% to 32.3%) | 11 | 14.9% (0.0% to 35.9%) | 88 | 25.0% (16.0% to 34.0%) |
| Q2 2020 | 233 | 58 | 24.9% | 57 | 22.8% (11.9% to 33.7%) | 42 | 19.0% (7.1% to 30.9%) | 48 | 27.1% (14.5% to 39.7%) |
| Q3 2020 | 219 | 49 | 22.4% | 41 | 12.2% (2.2% to 22.2%) | 43 | 23.3% (10.7% to 35.9%) | 9 | 0.0% (0.0% to 0.0%) |
| Q4 2020 | 230 | 55 | 23.9% | 48 | 6.3% (0.0% to 13.2%) | 40 | 10.0% (0.7% to 19.3%) | 14 | 28.6% (4.9% to 52.3%) |
| Q1 2021 | 283 | 79 | 27.9% | 73 | 11.0% (3.8% to 18.2%) | 74 | 14.9% (6.8% to 23.0%) | 12 | 41.7% (13.8% to 69.6%) |
| Q2 2021 | 232 | 69 | 29.7% | 61 | 4.9% (0.0% to 10.3%) | 64 | 17.2% (8.0% to 26.4%) | 10 | 35.4% (5.8% to 65.0%) |
| Q3 2021 | 370 | 85 | 23.0% | 78 | 6.4% (1.0% to 11.8%) | 78 | 6.4% (1.0% to 11.8%) | 19 | 13.0% (0.0% to 28.1%) |
| Q4 2021 | 354 | 86 | 24.3% | 78 | 17.9% (9.4% to 26.4%) | 35 | 28.6% (13.6% to 43.6%) | 20 | 15.0% (0.0% to 30.6%) |
| Q1 2022 | 363 | 91 | 25.1% | 87 | 14.9% (7.4% to 22.4%) | 68 | 16.2% (7.4% to 25.0%) | 68 | 11.8% (4.1% to 19.5%) |
| Q2 2022 | 405 | 89 | 22.0% | 89 | 20.2% (11.9% to 28.5%) | 80 | 17.5% (9.2% to 25.8%) | 80 | 15.0% (7.2% to 22.8%) |
| Q3 2022 | 565 | 99 | 17.5% | 96 | 11.5% (5.1% to 17.9%) | 87 | 16.1% (8.4% to 23.8%) | 91 | 14.3% (7.1% to 21.5%) |
| Q4 2022 | 661 | 107 | 16.2% | 102 | 5.9% (1.3% to 10.5%) | 85 | 9.4% (3.2% to 15.6%) | 101 | 7.9% (2.6% to 13.2%) |
| Q1 2023 | 485 | 99 | 20.4% | 93 | 10.8% (4.5% to 17.1%) | 93 | 18.3% (10.4% to 26.2%) | 88 | 13.6% (6.4% to 20.8%) |
| Q2 2023 | 382 | 76 | 19.9% | 50 | 10.0% (1.7% to 18.3%) | 67 | 10.4% (3.1% to 17.7%) | 59 | 11.9% (3.6% to 20.2%) |
| Q3 2023 | 486 | 104 | 21.4% | 101 | 4.0% (0.2% to 7.8%) | 95 | 6.3% (1.4% to 11.2%) | 96 | 7.3% (2.1% to 12.5%) |
| Q4 2023 | 468 | 97 | 20.7% | 88 | 8.0% (2.3% to 13.7%) | 88 | 12.5% (5.6% to 19.4%) | 83 | 15.7% (7.9% to 23.5%) |
| **Total** | **6,134** | **1,344** |  |  |  |  |  |  |  |

| **S4: Detailed Vancomycin, Linezolid, and Teicoplanin consumption and % resistance exhibited by Staphylococcus spp. in the ICU from Jan 2020 to Dec 2023 by semesters** | | | | | | | | | |
| --- | --- | --- | --- | --- | --- | --- | --- | --- | --- |
|  |  |  |  | Vancomycin | | Linezolid | | Teicoplanin | |
| Semester | Total isolates | Number of *Staph.* spp. isolates | *Staph.* spp. Isolation rate | n | % R  (95% CI) | n | % R  (95% CI) | n | % R  (95% CI) |
| S1 2020 | 356 | 78 | 21.9% | 73 | 34.2% (23.3% to 45.1%) | 34 | 41.2% (24.7% to 57.7%) | 63 | 34.9% (23.1% to 46.7%) |
| S2 2020 | 167 | 36 | 21.6% | 32 | 6.3% (0.0% to 14.7%) | 30 | 20.0% (5.7% to 34.3%) | 8 | 12.5% (0.0% to 35.4%) |
| S1 2021 | 193 | 49 | 25.4% | 47 | 8.5% (0.5% to 16.5%) | 48 | 33.3% (20.0% to 46.6%) | 8 | 38.8% (5.0% to 72.6%) |
| S2 2021 | 397 | 85 | 21.4% | 80 | 16.3% (8.2% to 24.4%) | 57 | 19.3% (9.1% to 29.5%) | 14 | 7.1% (0.0% to 20.6%) |
| S1 2022 | 398 | 85 | 21.4% | 81 | 25.9% (16.4% to 35.4%) | 70 | 22.9% (13.1% to 32.7%) | 71 | 16.9% (8.2% to 25.6%) |
| S2 2022 | 708 | 90 | 12.7% | 85 | 11.8% (4.9% to 18.7%) | 78 | 21.8% (12.6% to 31.0%) | 85 | 17.6% (9.5% to 25.7%) |
| S1 2023 | 444 | 86 | 19.4% | 71 | 15.5% (7.1% to 23.9%) | 80 | 23.8% (14.5% to 33.1%) | 74 | 13.5% (5.7% to 21.3%) |
| S2 2023 | 460 | 85 | 18.5% | 79 | 5.1% (0.2% to 10.0%) | 79 | 11.4% (4.4% to 18.4%) | 76 | 13.2% (5.6% to 20.8%) |
| **Total** | **3123** | **594** |  |  |  |  |  |  |  |
